# Taking Care of an Adolescent and Young Adult Cancer Survivor: A Systematic Review of the Impact of Cancer on Family Caregivers

**DOI:** 10.3390/ijerph20085488

**Published:** 2023-04-12

**Authors:** Maria Carolina Neves, Ana Bártolo, Judith B. Prins, Célia M. D. Sales, Sara Monteiro

**Affiliations:** 1 Center for Psychology at the University of Porto, Faculty of Psychology and Education Sciences, University of Porto, 4200-135 Porto, Portugal; 2 I2P—Portucalense Institute of Psychology, 4200-072 Porto, Portugal; 3 RECI—Research in Education and Community Intervention, Piaget Institute—ISEIT/Viseu, 3515-776 Viseu, Portugal; 4 CINTESIS@RISE, Department of Education and Psychology, University of Aveiro, 3810-193 Aveiro, Portugal; 5 Department of Medical Psychology, Radboud University Medical Centre, 6500 HB Nijmegen, The Netherlands; 6 Departament of Social Sciences and Management, Open University, 1269-001 Lisboa, Portugal; 7 Center for Global Studies, Open University, 1269-001 Lisboa, Portugal

**Keywords:** adolescent, young adult, AYA, caregiver, cancer, survivor, psychology

## Abstract

Research usually investigates adolescents and young adults (AYA) with cancer in combination with younger and older cancer patients and survivors. However, AYAs with cancer are a unique group, and their caregivers’ experience may also differ from other caregivers of cancer survivors. This systematic review aims to understand the impact of a cancer diagnosis on family caregivers, comparing the experience of caregivers of AYA childhood cancer survivors (AYA CCS) and caregivers of AYA with cancer. Relevant studies were identified through PubMed, Scopus, and Web of Science databases, and their quality was assessed using the Joanna Briggs Institute’s critical appraisal checklists. Sixteen studies (17 reports) met the inclusion criteria. Findings were synthesized separately for caregivers of AYA CCS and caregivers of AYA with cancer. Results showed that caregivers in both groups experienced high distress after the diagnosis. Partners of AYAs with cancer experienced diminished quality of life (QoL) and over half reported moderate to high fear of cancer recurrence (FCR). Findings indicated that cancer negatively impacts family caregivers, regardless of the patient’s age at diagnosis. However, findings are heterogeneous, and most do not focus on QoL or FCR. More research is needed on the impact of cancer among these family caregivers.

## 1. Introduction

Family caregivers are essential for cancer patients and survivors, but the demands placed on them can exceed their resources [1]. Approximately 20% of caregivers of adult patients reported high distress and burden, even after treatment. The burden and distress experienced by the family caregiver at baseline were predictors of their well-being after the patients’ treatments ended [2]. Additionally, the incidence of distress was higher when the patient reported high distress and poor physical functioning and when the caregiver reported a high burden and low social support [1]. Additionally, among caregivers of pediatric and caregivers of adult patients, research showed a bi-directional and interdependent relationship between the distress experienced by the caregiver and the distress experienced by the cancer patient [3]. This shows that taking care of adult cancer patients can negatively impact their caregivers.

Regarding caregivers of young people with cancer, the literature seems to focus more on caregivers of pediatric patients younger than 18 at diagnosis. Parents of these patients experienced a high emotional reaction (uncertainty, anxiety, depressive symptoms, and posttraumatic stress symptoms) at the time of diagnosis [4], with 40–42% of the families experiencing a moderate to very intense negative impact upon a cancer diagnosis [5]. For most parents, this emotional reaction decreases with time. However, for a subsample of parents, high levels were reported even five years or more after the diagnosis [4]. Among parents of childhood cancer survivors (CCS), 8.8–23% reported high psychological distress and posttraumatic stress symptoms, as well as feelings of guilt, anger, self-blame, and fear of cancer recurrence (FCR) [6].

The distress experienced by parents impacts not only their well-being but also their children’s. A positive association was found between parental distress and child distress [7] and quality of life (QoL) [8]. The parent perception of their child’s symptoms moderated a stronger association between parent and child distress. More so than the child’s self-report. This suggests that parental distress may influence their perception of their child’s well-being [7]. Disruptions in family dynamics have also been reported. A cancer diagnosis negatively impacted the relationships between the sibling of the patient and their parents, and even the caregivers’ spouse/partner relationships [5]. Thus, the impact of the diagnosis among caregivers of pediatric patients seems to be broad, impacting the nuclear family.

Cancer affects nearly six times more adolescents and young adults (AYAs; 15–39 years at diagnosis) than children (<15 years at diagnosis) [9]. The existing literature has frequently combined the study of the AYA group with that of pediatric and/or adult cancer patients and survivors. Additionally, when searching for studies about AYAs, these usually include childhood cancer survivors who have grown up into AYAs. However, AYAs are a unique group with physiological, developmental, and societal characteristics and challenges distinct from those of younger and older cancer patients. This limits the knowledge we have of the specific needs of AYAs with cancer [10]. Some studies, like the Childhood Cancer Survivor Study [11], are focused on AYAs who are childhood cancer survivors (hereafter referred to as AYA CCS). This means they had a cancer diagnosis when they were younger than 15 years old and were more dependent on their caregivers. At the time they participated in a study, they were already cancer survivors in their adolescence or young adulthood (between 15 and 39 years old). However, study results may present several specificities when the focus of the study is on adolescents and young adults diagnosed with cancer between the ages of 15 and 39 (hereafter referred to as AYA with cancer) [10]. Thus, the importance of differentiating the challenges of a diagnosis at this developmental transition stage is highlighted.

According to the theory of emergent adulthood [12], adolescence and young adulthood are periods of change and exploration. AYAs with cancer are in a challenging phase where biological, physiological, psychological, developmental, and societal changes occur [10]. Confrontation with a cancer diagnosis and treatments during adolescence and young adulthood can disrupt their life and lead to additional issues. Some examples are a premature confrontation with mortality, changes in their body, increased dependency, and disruption in social life, school, and employment [13]. Compared to AYA CCS treated at a younger age, AYAs with cancer have a greater cognitive capacity to understand the severity of their diagnosis at the time [13]. Therefore, a cancer diagnosis possibly has a different impact depending on the developmental stage at which it is diagnosed.

In line with this, it is also possible that their caregivers’ experience regarding the psychosocial impact (e.g., anxiety, depression, quality of life, among others) of caring for a young person with cancer is different when caring for an AYA CCS and an AYAs with cancer. However, studies have yet to compare the experience of these two groups of caregivers. Ljungman and colleagues [6] systematically reviewed the positive and negative long-term psychological effects of a cancer diagnosis and treatments on parents of AYA CCS. They found that parents of AYA CCS usually report psychological distress within a normal range. However, a subgroup of parents of AYA CCS reports high distress and post-traumatic stress symptoms. Anger, guilt, self-blame, and FCRwere also reported. As for positive effects, some parents of AYA CCS stated that their marital relationship was strengthened by the cancer experience, other relationships also improved, and their values changed. However, a subgroup still reported marital difficulties. Nevertheless, there is no guarantee that these findings reflect the experience of caregivers of AYAs with cancer. More research is needed to know if their experiences are comparable. It is essential to understand if the experiences of caregivers of AYA CCS and caregivers of AYA with cancer are similar or different, and if so, in what aspects. This will ensure that the care provided to each group of caregivers tackles their specific needs. In addition, the literature is not yet systematized regarding caregivers of AYA with cancer.

To fill these gaps, this systematic review aims to understand the impact of a cancer diagnosis on family caregivers by comparing the experience of caregivers of AYA CCS (AYA CCS were 0–14 years at diagnosis and 15–39 at recruitment) and caregivers of AYA with cancer (AYA patients were 15–39 years at diagnosis and recruitment). Based on this objective, the following research questions are proposed:

(i) What is the psychological impact (e.g., FCR, distress, anxiety, depression, QoL) reported by caregivers of AYA CC and caregivers of AYA with cancer, and its prevalence?

(ii) What are the predictors and mediator factors of the psychological impact experienced by caregivers of AYA CCS and caregivers of AYA with cancer?

(iii) What are the differences between the impact reported by caregivers of AYA CCS and caregivers of AYA with cancer?

## 2. Materials and Methods

This systematic review was previously registered at PROSPERO (number CRD42020219201) and conducted following the Preferred Reporting Items for Systematic Review and Meta-Analyses (PRISMA) 2020 guidelines [14].

### 2.1. Literature Search

The authors systematically searched PubMed, Scopus, and Web of Science electronic databases on 24 November 2020 for the first time. The search was re-run on 14 March 2022 to identify possible further reports published between 25 November 2020 and 14 March 2022. We restricted the search to English records published from 2010 onward. Additionally, by searching the references of the included studies, a manual search was performed to identify potentially relevant reports that could have been missed in the database search.

**Figure 1:**
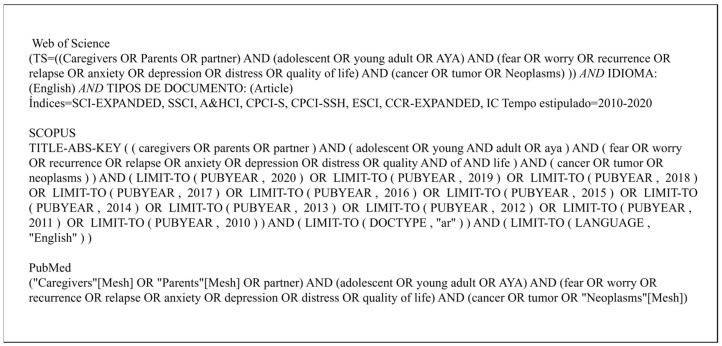
Search strategy used for each database.

The search terms included keywords related to the population (caregiver, parent, partner, adolescent, young adult, AYA), outcomes (fear, worry, recurrence, relapse, anxiety, depression, distress, QoL), and cancer diagnosis (cancer, tumor, neoplasm). The functions “OR” and “AND” were also included. Figure 1 outlines the search strategy used for each database.

### 2.2. Inclusion and Exclusion Criteria

Studies were considered for inclusion if they were (a) written in English; (b) published in peer-review journals from 2010 onward; (c) using quantitative and/or qualitative methods; (d) with a sample of family caregivers of AYAs CCS or family caregivers of AYA with cancer and (e) reporting psychological outcomes (distress, anxiety, depression, QoL and/or FCR). Although studies may include caregivers of cancer survivors in different age groups, we only considered them if family caregivers of AYAs with cancer were analyzed separately. Conference papers, commentaries, editorials, dissertations, review papers, and case studies were excluded, as well as those involving caregivers of adults with cancer.

### 2.3. Data Extraction

After removing duplicate studies, the titles and abstracts of potential records were reviewed for eligibility by the first author (MCN) using the Rayyan website [15] and considering the inclusion and exclusion criteria pre-established by the review team. Next, the author independently evaluated the full-text report and excluded those not meeting the inclusion criteria. The reasons for exclusion were registered. Then, this author manually screened the reference lists of the included studies for any additional study. Any doubts were discussed and resolved by consensus between the coauthors throughout the process.

The author MCN extracted the data of each included study concerning the source of the study (authors, year of publication, title, design, country), the aim of the study, the instrument used to assess psychological adjustment, participants’ characteristics for both the caregiver and the AYA with cancer (sample size, type of caregiver, age of caregiver, age of AYA at diagnosis, age of AYA at recruitment, sex, education, employment, marital status, cancer diagnosis, time since diagnosis, treatment status), and the main conclusion/findings of the study for each psychological domain.

### 2.4. Quality Assessment

The quality of the included studies was assessed by the first author using the Joanna Briggs Institute’s (JBI) Critical Appraisal Checklist for both analytical cross-sectional studies and qualitative research [16,17]. These checklists consist of eight to ten items that assess the paper’s quality using qualitative descriptors (yes, no, unclear, or not applicable).

### 2.5. Data Synthesis

The findings were synthesized separately considering the caregiver group (caregiver of AYA CCS vs. caregiver of AYA with cancer). The study type (quantitative vs. qualitative) is also stated separately in each group to provide a better overview. We only presented multivariate analyses in quantitative studies where both univariate and multivariate analyses were available.

## 3. Results

### 3.1. Study Selection

**Figure 2:**
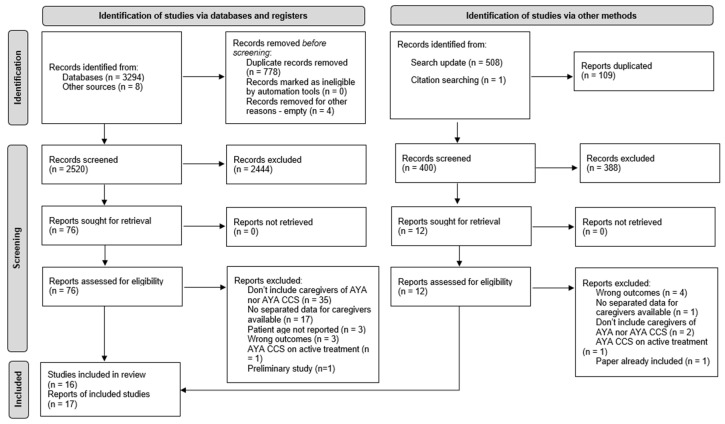
PRISMA 2020 flow diagram.

A flow diagram with the literature search is presented in Figure 2. As shown, the database search identified 3294 records. Eight records from other sources were added. Of these 3302 records, four appeared as “empty” (i.e., with no information), and 778 were identified as duplicates. From these, 2520 records were eligible for the title and abstract screening. Most records were focused on other populations (i.e., pediatric cancer patients or adults with cancer) and some on other outcomes. Thus, we retrieved 76 reports for full-text screening. Of these, 36 did not include family caregivers of AYA CCS nor AYA with cancer; 17 did not have available data for caregivers separately; in three studies, the age of the cancer patient was not reported, not allowing us to understand whom the caregiver was caring for; three focused on other outcomes; and one was a preliminary study of an included report. In addition, two reports belonged to the same study. However, these reports presented different research questions and were included in the review. The authors included those findings separately but considered the sociodemographic and clinical data as one.

During the update search, we identified 508 additional records. Of these, 109 were duplicates. Three hundred and ninety-nine records were eligible for the title and abstract screening. Among these, we only retrieved 12 reports for full-text screening. Four focused on other outcomes, one did not have separate data for the caregiver, two were not focused on caregivers of AYA with cancer or AYA CCS, one was focused on AYA CCS who were still on active treatment, and one report was a duplicate of a study previously included. This led to the inclusion of two additional studies. Manual search found another study. We included a total of 16 studies (17 reports).

### 3.2. Characteristics of the Included Studies

We included sixteen studies in this systematic review. Of these, seven studies were focused on the psychological impact of cancer on caregivers of AYA CCS [18,19,20,21,22,23,24], and nine studies on caregivers of AYA with cancer [25,26,27,28,29,30,31,32,33,34].

Among those that focused on caregivers of AYA CCS (*n* = 7), one study had a qualitative design, with focus groups being used for data collection [18], while six studies had a quantitative design [19,20,21,22,23,24]. Most quantitative studies were cross-sectional [19,20,22,23,24], with one being longitudinal [21]. As for data collection methods, studies recruited participants in person (*n* = 2) [19,22] and by mail (*n* = 4) [20,21,23,24]. Most of the studies were carried out in the USA (*n* = 4) [19,20,22,23], and some in Canada (*n* = 1) [24], Ireland (*n* = 1) [18], and Belgium (*n* = 1) [21]. All these studies focused on parents, primarily mothers (46.4–96.6%). Only one study included more fathers (53.6%) than mothers [19]. As for the sample size, it varied between 18 [18] and 224 [21]. Parents’ mean ages ranged from 43.62 to 51.98 years old, and most had completed higher education. Most parents cared for AYA CCS with a wide variety of diagnoses, and 1 study focused on parents of brain tumor patients [19].

As for those studies with a focus on caregivers of AYA with cancer, three studies used a qualitative design [19,25,26,28,32,34] with data collected using semi-structured interviews, and six studies used a quantitative design and used a variety of methods to collect data [20,21,22,23,24,25,26,27,28,29,30,31]. These quantitative studies included questionnaires being sent by mail (*n* = 2) [26,27,30], questionnaires being delivered in person but answered by the participants at home (*n* = 1) [28], and web-based surveys (*n* = 2) [25,27,31,33]. One study allowed the participants to answer online or using pen and paper [29]. Six studies were cross-sectional [20,21,22,24,25,26,27,28,30,31], and 1 was a longitudinal study [29]. Like before, most of them were carried out in the USA (*n* = 6) [20,21,23,25,26,27,29,31,33]. However, studies from Australia (*n* = 1) [30], France (*n* = 1) [28], and China (*n* = 1) [25] were also found. As for the caregiver type, most of these studies focused on parents (*n* = 6) [19,23,24,25,27,28,29,30,31,33,34], while some had partners (*n* = 2) as their focus [26,27,28] and one study included both parents and partners [32]. The sample size varied between 8 [32] and 491 [28] caregivers, mainly including females (50–100%). However, one study focused only on male partners [28]. These caregivers’ ages ranged from 24–65 years old, and most completed high school or higher education, although most studies did not report education data. As for cancer diagnosis, most studies focused on caregivers of AYAs with a wide variety of diagnoses, and two focused on caregivers of breast cancer patients [26,27,28].

The characteristics of the included studies are presented in Table 1 and Table 2.

### 3.3. Study Quality

Descriptions of the critical appraisal are shown in Table 3. Both qualitative and quantitative studies meet nearly all the JBI criteria. However, most qualitative studies did not include a statement locating the researcher culturally or theoretically, nor did they state the researcher’s influence on the research. As for quantitative studies, only 5 out of 13 identified confounding factors and strategies to deal with them. For three studies, it was unclear if the authors of these studies measured the outcomes with instruments validated for their country.

### 3.4. Distress

#### 3.4.1. Caregivers of AYA CCS

The quantitative research results included in this review on levels of distress and caregiver characteristics have shown diverse findings. One study found that parents have similar levels of distress as those reported by survivors and their siblings (F(2, 75) = 5.32, *p* < 0.01) [24]. Another study found that parents report more cancer-related worries than survivors (mothers: t(113) = −6.85, *p* < 0.001; fathers: t(81) = −6.26, *p* < 0.001) [21]. The distress levels reported by parents of AYA CCS also seem lower than those of parents of healthy peers (*p* = 0.043) [22]. Additionally, mothers also reported more depressive symptoms when compared to AYA CCS (t(112) = −2.28, *p* = 0.025) [21].

High distress levels have been associated with characteristics of both the AYA CCS and the parents. More specifically, parents experienced high distress when both the child (r = 0.22, *p* < 0.01) and the parent (r = 0.52, *p* < 0.001) reported the child had less positive and more negative adjustment outcomes (more internalizing problems) [22]. High parental distress was also found when the child reported less personal adjustment (r = −0.19, *p* < 0.01) and when the parents reported less child adaptive skills (r = −0.30, *p* < 0.001) [22]. Additionally, high distress levels in parents were associated with both parents (r = −0.24, *p* < 0.01) and survivors (r = −0.21, *p* < 0.05) reporting less positive parent-child relationships [22]. Furthermore, cancer-related worries positively correlated to depressive symptoms (r = 0.33, *p* < 0.001) among parents. In addition, married mothers and those living together experienced lower depressive symptoms at 1-year follow-up compared to baseline levels (β = −0.24, *p* = 0.013) [21].

Evidence suggests that Hispanic parents living in the United States were particularly vulnerable, with studies indicating higher depressive symptoms in Hispanic parents compared to non-Hispanic parents [20,23]. This is true for Hispanic parents born in the United States and those born in a foreign country [20]. Their depression levels can be twice as high (48% of Hispanic parents vs. 25% of non-Hispanic parents, *p* = 0.002) [20]. Additionally, being a Hispanic parent with lower income, high perceived stress, and perceiving that their child had low psychological functioning was associated with higher depression levels (F(5130) = 59.75, R^2^ = 0.71, *p* < 0.0001) [20].

Some studies investigated potential predictors, mediators, and moderators of the distress parents experience. Being younger, using less avoidant coping, and experiencing more satisfaction with life were found to predict less parental distress (F(3, 22) = 7.69, R^2^ = 0.51, *p* < 0.001) [24]. Among fathers only, a long time since diagnosis predicted lower cancer-related worries at 1-year follow-up (β = −0.32, *p*< 0.001) [21]. The perceived quality of the parent-child relationship reported by each member seems to be an important variable. It mediated the associations between parental distress and adjustment outcomes of both parents and survivors (β = 0.10, *p* = 0.05). More specifically, more parental distress was associated with less parental care and more overprotection reported by survivors. This led to poor adjustment and high internalizing problems reported by survivors. More parental distress also led to parents feeling less attached to their child and more frustrated with their relationship with their child. This led to parents reporting that the survivors had more internalizing problems and less adaptive skills (β = −0.11, *p* = 0.02) [22]. Additionally, being Hispanic moderated the relationship between parental stress and survivors’ mental health, meaning that for Hispanic parents only, high parental stress was associated with high levels of depressive symptoms (β = 0.390, *p* = 0.002) and low QoL in survivors (β = −0.445, *p* < 0.001) [23].

As for qualitative evidence of the included studies, even though parents experience high distress during their child’s diagnosis and treatments, they reported being particularly vulnerable in the post-treatment phase. This phase was the first time they could reflect, start to process what had happened, and focus on their well-being. Despite being anticipated, the end of treatments also brought mixed emotions. Some parents described mental health crises, which were considered a consequence of the lack of psychological support available. Parents wanted this kind of support to be available from the time of diagnosis and open to all family members [18].

#### 3.4.2. Caregivers of AYA with Cancer

Quantitative evidence of the included studies suggests that approximately one-third of the mothers have high distress levels [29]. More than one-third of the parents experienced moderate to severe depression (28–31.8%) [24,25,30,31] and anxiety (28%) [30], and an additional 26% reported mildly elevated mental health difficulties [30]. Interestingly, when the AYAs with cancer lived with their parents, the latter reported higher anxiety symptoms (r = 0.40, *p* = 0.001) [33].

As for partners of breast cancer patients, 20.53% report high to very high anxiety levels, similar to those reported by AYAs with cancer (22.31%). These were notably higher in the chemotherapy group compared to other groups (receiving Trastuzumab, receiving hormonal therapy, and on follow-up) (β = 3.50, *p* < 0.05). However, for depressive symptoms, partners report nearly twice as many depressive symptoms as AYAs with cancer (47% vs. 28%) [28].

Other life stressors, having plans for the future, and perceived impact on the broader family were predictors of parental distress (F(5, 183) = 10.91, R^2^ = 0.23, *p* < 0.001) [30]. Additionally, high maternal distress at baseline predicted worse AYA with cancer resilience at 3–6 months follow-up (β = −0.48, *p* = 0.043) [29].

Cognitive processing and FCR together seem to be important mediators of the relationship between social constraints (i.e., more difficulty disclosing negative thoughts, concerns, and feelings to others) and depressive symptoms. More specifically, parents that experienced high social constraints seem to inhibit their cognitive processing of the situation (i.e., led to more intrusive thoughts and avoidance) and have high FCR levels, which in turn led to high depressive symptoms (F(4, 61) = 16.30, R^2^ = 0.52, *p* < 0.001) [31]. Moreover, higher intolerance of uncertainty led to lower subjective well-being by parents, mediated by high insomnia and depressive symptoms (F(5, 53) = 8.09, R^2^ = 0.43, *p* < 0.0001) [33].

Comparable to quantitative evidence of the included studies, a qualitative study also found that both parents and partners experience high anxiety and depression. These feelings were enhanced by their sense of responsibility for their loved ones, leading to them feeling guilty. Particularly partners found it difficult to remain positive [32]. In one-child families, parents were also worried about the treatment’s effectiveness and side effects. They felt anxious and cried when alone. Two mothers had suicidal thoughts, with one of them having attempted suicide. However, they constantly tried to suppress how they felt by trying to remain calm and optimistic when in front of their child [25].

### 3.5. Quality of Life

#### 3.5.1. Caregivers of AYA CCS

Only one included study focused on the QoL of parents of AYA CCS. It found that parents’ QoL was similar to the general population (physical health: mean = 48.5; 95% CI = 44.6–52.4, *p* = 0.46; mental health: mean = 47.8; 95% CI = 44.0–51.7, *p* = 0.27). Nonetheless, some participants scored 0.5 standard error below the mean for physical (39%) and mental (42%) health, being at risk for adverse physical and mental health. Adverse physical and mental health effects were associated with a greater perceived impact of the diagnosis at the family level (physical health: OR = 1.18; 95% CI = 1.03–1.35; mental health: OR = 1.13; 95% CI = 1.01–1.27), with the presence of a history of depression in the survivor (physical health: OR = 1.12; 95% C = 1.02–1.24; mental health: OR = 1.00; 95% CI = 1.00–1.20), and with a history of cancer recurrence (OR = 12.5; 95% CI = 1.20–130.6). Adverse parental mental health was also associated with parental unresolved anger/sorrow (OR = 3.04; 95% CI = 1.01–9.14), long-term uncertainty, and when the child had speech/language problems (OR = 19.20; 95% CI = 1.84–199.94). As for adverse physical health, it was related to the parent perceiving the child had a better cognitive function (OR = 1.04; 95% CI = 1.00–1.07) and perceiving the child had emotional/behavioral problems (OR = 1.25; 95% CI = 1.01–1.56). However, both better physical and mental health of parents were associated with greater emotional resources (physical health: OR = 0.35; 95% CI = 0.12–0.99; mental health: OR = 0.35; 95% CI = 0.12–1.00) and when parents perceived an improvement in their child’s relationship with their peers (physical health: OR = 0.90; 95% CI = 0.82–0.98; mental health: OR = 0.88; 95% CI = 0.80–0.98) [19].

#### 3.5.2. Caregivers of AYA with Cancer

The results of the quantitative included studies showed that younger partners reported better physical (ES = −0.57) and sexual functioning (ES = −0.61) and lower sexual difficulties (ES = −0.82) than older partners. However, younger partners also experienced lower marital satisfaction (ES = 0.39), more hyperarousal (ES = −0.33), and overall worst QoL (ES = 0.43). As for potential predictors, greater physical functioning, fewer depressive symptoms, higher marital satisfaction, higher parenting satisfaction, and more personal resources (like social support) seem to predict an overall better QoL for younger partners (F(5, 195) = 35.05; R^2^ = 0.47; *p* < 0.001) [27].

Regarding qualitative studies included in this review, caregivers considered improving doctor-patient communication could enhance their QoL, mainly through increased and accessible communication consistent with printed materials and delivered in multiple ways. Some ways reported include face-to-face communication, pamphlets, and ongoing written reports. They considered this could improve their QoL by allowing them to understand the information they would not be able to retain otherwise. A few caregivers also mentioned a desire for more research-based treatment options and more directiveness from providers [32].

### 3.6. Fear of Cancer Recurrence

#### 3.6.1. Caregivers of AYA CCS

Qualitative research included found that some parents were hypervigilant for any symptom of recurrence during follow-up, while at the same time, they tried not to smother their child. FCR, fear for their child’s survival, fear that they might miss an early sign or symptom, fear over a lack of access to services or supports, fear relating to their child’s readjusting socially, and fear relating to the late effects of treatment could explain why parents found it so difficult to withdraw from follow-up care [18].

#### 3.6.2. Caregivers of AYA with Cancer

One quantitative study included in this review found that over half of the partners (53.6%) experienced moderate to high FCR levels. However, this did not differ much from those reported by breast cancer survivors included in the study, with 52.3% also experiencing moderate to high FCR. Findings also indicate that cognitive processing mediates the relationship between social constraints and FCR (F(3215) = 27.917, R^2^ = 0.280, *p* < 0.001). This means that partners who experience more constraints to express themselves have more difficulty cognitively processing the trauma, leading to higher FCR levels [26].

As for qualitative evidence included, parents described how they experienced FCR daily within the first-year post-treatment, especially when the adolescent had any symptoms. “*When he gets sick, a lot of things go through your mind. Fear, everything*”. They also did not expect it would disappear soon, and seeing other families face relapse led to additional fear. These parents reported feeling unprepared for a possible relapse, not knowing what signs or symptoms to watch out for. As for strategies to manage this fear, parents reported being diligent (watching out for physical and emotional changes and symptoms, seeking medical attention when symptoms appeared, asking how their child was feeling), more proactive about their child’s care (by trying to be informed, making lifestyle changes, talking about their concerns), praying (having faith, letting God take care of their fears and concerns) and taking it day by day (trying not to think too far into the future) [34]. Parents also believed the risk of recurrence would persist and viewed the future pessimistically [25].

**Figure 3:**
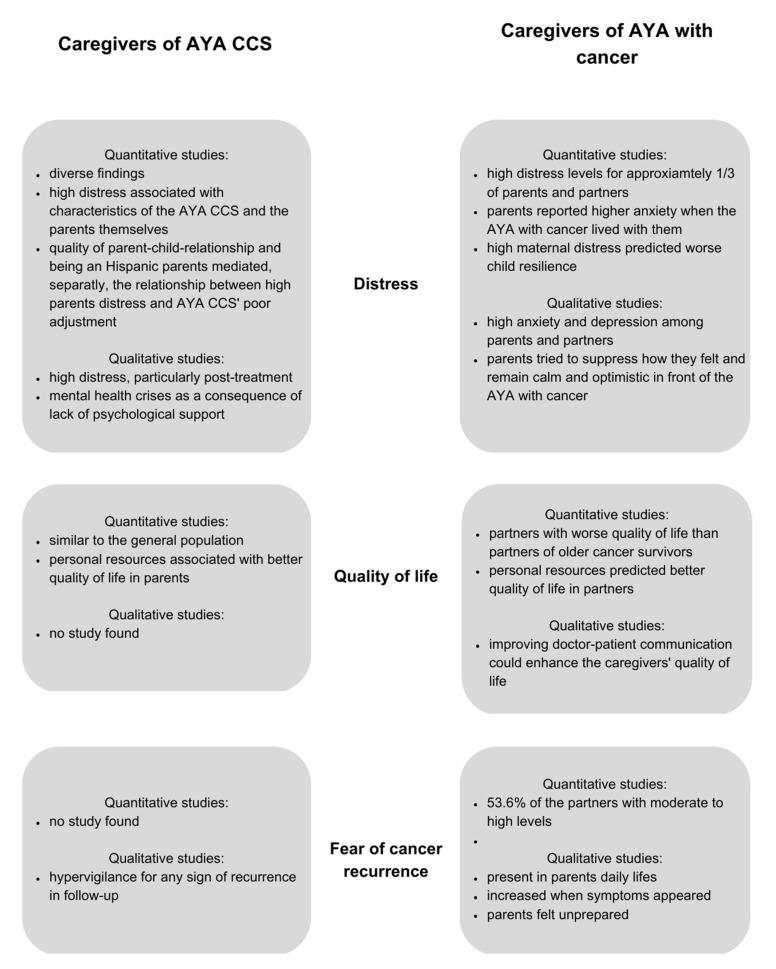
Summary of the impact of a cancer diagnosis on caregivers of AYA CCS and caregivers of AYA with cancer.

A summary of the results can be found in Figure 3.

## 4. Discussion

This systematic review aimed to understand the impact of a cancer diagnosis, comparing the experience of caregivers of AYA CCS and caregivers of AYAs with cancer. Research usually investigates AYAs with cancer in combination with younger and older cancer patients and survivors. However, AYAs with cancer are a unique group. It is essential to understand better how similar or different their family caregivers’ experiences are, so their care can be focused on things that are important to them. This systematic review found limited literature on caregivers of AYA CCS and caregivers of AYA with cancer. Findings indicate that a cancer diagnosis can have a negative impact on some caregivers of AYAs with cancer, regardless of the patient’s age at diagnosis. However, findings are heterogeneous, and most included articles focused on distress. Only a few provided information regarding these caregivers’ QoL and FCR levels.

Parents of AYA CCS, as well as parents and partners of AYAs with cancer, experienced high distress following a cancer diagnosis. Very few studies reported prevalence levels. These showed that approximately 1/3 of parents of AYAs with cancer experienced high distress. Additionally, 20.53% of partners of AYA breast cancer patients and 28% of parents of AYA with cancer had high anxiety levels. As for high depression, it was reported by 47% of the partners of breast cancer survivors and 28–31.8% of the parents of AYA with cancer. High distress had also been found among family caregivers of adult cancer survivors [1,35] and parents of pediatric cancer patients [6]. This suggests that high distress is a common and universal caregiver experience, independent of the patient’s age at diagnosis. No study was found with a focus on distress among partners of AYA CCS, constituting a research gap. Health professionals must pay attention not only to parents of AYA CCS and AYA with cancer but also to their partners.

Some parents seem to be at risk for mental health problems. First, Hispanic parents of AYA CCS living in the United States seem to be particularly vulnerable to depression symptoms when compared to non-Hispanic parents. Among the included studies, Hispanic parents of AYA CCS living in the United States had a lower income, lower education, and less health insurance than non-Hispanic parents. This is in line with what was previously found, where more financial stress was related to high depression, especially in low-income populations [36]. These Hispanic parents may be experiencing more social and economic stressors than non-Hispanic parents, leading to more distress. A study by Casillas and colleagues [37] also found that Hispanic parents living in the United States felt isolated from their extended family and the community. They also hesitated to share their experience regarding their child’s cancer with their family and friends. This isolation and lack of sharing their concerns could potentially enhance the depressive symptoms experienced by these parents. This shows that it is crucial to pay more attention to Hispanic parents and possibly other cultural minorities, as well as people in a more socially fragile situation.

Second, one study found that mothers of AYA CCS are at risk for higher distress and depression than fathers and AYA CCS themselves. A meta-analysis of 58 studies about the prevalence of anxiety and depressive symptoms in patients of children with cancer [38] found heterogeneous results. Anxiety levels ranged from 5% to 65%, and depressive levels from 7% to 91%. Even though mothers had a higher prevalence of depression than fathers, this difference was not significant. These contrary results leave questions about the potentially higher risk of mothers for distress and depression. No study was found investigating the differences in distress levels between mothers and fathers of AYA. More research is needed to understand the risk for both groups of caregivers better.

Third, in one study, being married or living together seemed protective for mothers of AYA CCS, with their depressive symptoms decreasing at 1-year follow-up. A previous study interviewed parents of children with cancer after the end of treatments. They found that the diagnosis of cancer in a child caused changes in the entire family system. However, approximately 40% reported positive changes in their relationship with their partner. A strong relationship with their partner before the diagnosis significantly influenced their relationship during and after treatments. Their partnered relationship was also considered an enormous resource by approximately 70% of the parents in a lived relationship [39]. This could help explain why mothers of AYA CCS who are still married or living together 1 year after diagnosis experienced a decrease in depressive symptoms. Lastly, parents that lived with their AYA child with cancer reported high anxiety symptoms. More research is needed to know if these parents are at higher risk of experiencing anxiety.

Additionally, even though parents of AYA CCS reported QoL levels similar to the general population, the partners of AYA with cancer reported worse QoL than partners of older cancer survivors. This could suggest that partners of AYA may be at risk for adverse QoL. Partners of AYA with cancer probably did not expect to have to take care of their romantic partner at such a young age. Thus, the unexpected onset of a severe illness and this change in roles may contribute to their low QoL. A previous study found that caregivers of adults with cancer experience worse QoL during active treatment, which improves at follow-up [35]. This could explain why caregivers of AYA CCS, who have survived cancer, experience QoL levels like those of the general population. However, more studies are needed to understand these differences better. It would also be important to explore the QoL of parents of AYA with cancer, both in active treatment and follow-up phases.

Supported by the literature on caregivers of adults with cancer [35], it was also found that having more personal resources, like social support, was associated with better QoL in caregivers of AYA CCS and was a predictor of better QoL in partners of AYA with cancer with cancer. Social support was also a protective factor for distress among caregivers of pediatric patients [4]. Personal resources seem to play an important role in improving the QoL experienced by caregivers. Thus, it would be important that interventions include social support as one of their focuses. The intervention should preferably be delivered face-to-face, which has been found to have better results on interventions focused on improving the QoL of caregivers of children and adolescents with chronic conditions [40].

Finally, over half of the partners of AYA experienced moderate to high FCR levels. However, no study focused on the FCR levels experienced by parents of either AYA CCS or AYA with cancer. In recent years, more research has focused on the FCR experienced by cancer patients and their caregivers. A recent systematic review showed that approximately half of the caregivers of adults with cancer experienced FCR. Their levels were found to be similar and sometimes even higher than those reported by survivors [41]. It is crucial that research starts to focus on the FCR experienced by caregivers of children, adolescents, and young adults with cancer, so we can better understand how they feel about the possibility of recurrence and find ways to help families cope with this fear and uncertainty of the future.

Nevertheless, this review has some limitations. First, high heterogeneity of studies was found, and few studies were found, especially focused on QoL and FCR. This does not allow us to draw any solid conclusions regarding the impact of a cancer diagnosis on these caregivers. Second, the articles search was restricted to publications from 2010 onward and written in English. This could have excluded papers written before 2010 and/or written in another language. Lastly, the keywords used for the outcomes also limited the caregivers’ experiences to their distress levels, QoL and FCR. It is also possible that the diagnosis positively impacted some caregivers, though this review did not intend to explore this impact.

The findings of this systematic review alert us to the importance of studying the impact of a cancer diagnosis on caregivers of AYA with cancer, either diagnosed during childhood or adolescence and young adulthood. Future studies should prioritize the assessment of predictors of distress, QoL, and FCR on caregivers of AYA with cancer. Additionally, they could compare the experience of caregivers of AYA CCS and caregivers of AYA with cancer to better understand their similarities and differences. A systematic review focusing on the positive impact of cancer on caregivers is also critical to give us a broader view of the impact of a cancer diagnosis on caregivers of AYA with cancer.

## 5. Conclusions

The results of this review show that parents of AYA CCS, and parents and partners of AYA with cancer report high distress levels. Partners of AYA with cancer also seem to experience diminished QoL compared to partners of older survivors, with personal resources as a protective factor. Additionally, over half of the partners of AYA with cancer experience moderate to high FCR. Hispanic parents, mothers, and parents living with their AYA child with cancer are potentially at higher risk for mental health issues, while being married or living together is potentially protective for mothers. This suggests that a cancer diagnosis negatively impacts some family caregivers of AYAs with cancer similarly for caregivers of either AYA CCS or AYA with cancer. However, these results should be considered with caution since the evidence found is limited and the studies focused on QoL and FCR among caregivers of AYA CCS and caregivers of AYA with cancer are scarce. We hope this review adds to the literature that shows the importance of caregivers and family-centered care, highlighting gaps that need further exploration.

## Figures and Tables

**Table 1:** Summary of the characteristics of the included studies.

Author, Year	Country	Design	Data Collection Method	Type of AYA	Type of Caregiver	Caregiver: Measures
Barrett et al., 2020 [18]	Ireland	Qualitative	Focus group	AYA CCS	Parents	N/A
Buchbinder et al., 2017 [19]	USA	Quantitative	In-person	AYA CCS	Parents	QoL: Patient-reported Outcomes Measurement Information System (PROMIS) Global Health
Chen et al., 2022 [25]	China	Qualitative	Semi-structured interviews	AYA	Parents	N/A
Cohee et al., 2017, 2018 [26,27]	USA	Quantitative	Sent by mail	AYA	Partner	FCR: Concerns About Recurrence Scale (CARS) Depression: Center for Epidemiologic Studies—Depression Scale (CES-D) QoL: Index of Well-Being (IWB)
Congard et al., 2019 [28]	USA	Quantitative	In-person recruitment, but participants responded at home	AYA	Partner	Anxiety: State-Trait Anxiety Inventory (STAI) Depression: CES-D
Lau et al., 2020 [29]	USA	Quantitative	Online or pen and paper	AYA	Parents	Distress: Kessler Psychological Distress Scale 6 (K6)
McCarthy et al., 2016 [30]	Australia	Quantitative	Sent by mail	AYA	Parents	Distress: Kessler Psychological Distress Scale (K10)
Meeske et al., 2013 [20]	USA	Quantitative	Sent by mail	AYA CCS	Parents	Depression: CES-D
Mikrut et al., 2020 [31]	USA	Quantitative	Web-based survey	AYA	Parents	FCR; CARS Depression: Patient Health Questionnaire (PHQ-9)
Mishra et al., 2018 [32]	USA	Qualitative	Semi-structured interview	AYA	Parents and partners	N/A
Panjwani et al., 2020 [31]	USA	Quantitative	Web-based survey	AYA	Parents	Depression: PHQ-2 Anxiety: Generalized Anxiety Disorder Scale (GAD-7)
Prikken et al., 2022 [21]	Belgium	Quantitative	By mail	AYA CCS	Parents	Depression: CES-D
Schepers et al., 2019 [22]	USA	Quantitative	In-person	AYA CCS	Parents	Distress: Brief Symptom Inventory (BSI-18)
Slaughter et al., 2020 [23]	USA	Quantitative	Sent by mail	AYA CCS	Parents	Distress: PSS-4 QoL: Pediatric Quality of Life Questionnaire Depression: CES-D
Turner-Sack et al., 2016 [24]	Canada	Quantitative	Sent by mail	AYA CCS	Parents	Distress: BSI
Walker et al., 2020 [34]	USA	Qualitative	Semi-structured interview	AYA	Parents	N/A

**Table 2:** Summary of the characteristics of the included studies (continuation).

Author, Year	Caregiver: Sample Size	Caregiver: Sex	Caregiver: Age (Mean)	Caregiver: Education (Percentage or Years)	Caregiver: Marital Status (Percentage)
Barrett et al., 2020 [18]	18	F = 88.8%, M = 11.2%	Missing	Missing	Missing
Buchbinder et al., 2017 [19]	28	F = 46.4%, M = 53.6%	44 (7)	High school/GED or College/Grad School = 25 (89.3%)	Married or living with partner = 19 (67.9%)
Chen et al., 2022 [25]	14	F = 85.7%, M = 14.3%	53	Missing	Married = 12, Divorced = 2
Cohee et al., 2017, 2018 [26,27]	222/227	Missing	47.98 (7.2)/48.04 (7.04)	14.92 (2.6) years	Living with partner = 100%
Congard et al., 2019 [28]	491	M = 100%	43.28 (7.46)	14.88 (2.552) years	Missing
Lau et al., 2020 [29]	14	F = 100%	47	Missing	Missing
McCarthy et al., 2016 [30]	204	F = 90%, M = 10%	Missing	Missing	Married/partner = 154 (77%) Separated/Divorced = 23 (11.5%) Single = 23 (11.5%)
Meeske et al., 2013 [20]	Hispanic: 79; Non-Hispanic: 69	Hispanic: F = 90%, M = 10%; Non-Hispanic: F = 85%, M = 15%	Hispanic = 46.77 (6.19), Non-Hispanic = 51.79 (6.61)	Hispanic: Grade school (1–8 years) = 43(54%); Non-hispanic: High school = 20 (34%), Some college = 13 (22%), College/Grad school = 14 (24%)	Hispanic: Married = 59 (75%); Non-hispanic: Married = 38 (63%)
Mikrut et al., 2020 [31]	66	F = 92%, M = 8%	55.17 (7.02)	Missing	Married/in a long-term relationship = 80%
Mishra et al., 2018 [32]	8	F = 50%, M = 50%	24–65	College/Bachelor’s degree = 6	Married/living with partner = 6
Panjwani et al., 2020 [31]	59	F = 91%, M = 9%	55 (7.43)	Missing	Married/in a long term relationship = 80%)
Prikken et al., 2022 [21]	224	F = 59.4%, M = 40.6%	F = 49.4, M = 51.51	Higher education: F = 70%, M = 58%	Not married or living with a partner—F = 14%; M = 11%
Schepers et al., 2019 [22]	206	F = 89.9%, M = 12.1%	43.67 (7.03)	Missing	Missing
Slaughter et al., 2020 [23]	Hispanic: 68; Non-Hispanic: 61	Hispanic: F = 89.7%, M = 10.3%; Non-hispanic: F = 85.6%, M = 14.8%	Hispanic: 46.32 (6.07); Non-hispanic = 51.98 (6.33)	Missing	Missing
Turner-Sack et al., 2016 [24]	30	F = 96.6%, M = 3.4%	45.07 (5.64)	Graduated college/university = 20	Missing
Walker et al., 2020 [34]	30	F = 93%, M = 7%	47 (7)	Missing	Married = 23 (77%)

**Table 3:** Summary of the Critical Appraisal Criteria According to the Joanna Briggs Institute Statistics Assessment and Review Instruments.

	N
Qualitative research	
Is there congruity between the stated philosophical perspective and the research methodology?	2/4
Is there congruity between the research methodology and the research question or objectives?	4/4
Is there congruity between the research methodology and the methods used to collect data?	4/4
Is there congruity between the research methodology and the representation and analysis of data?	4/4
Is there congruity between the research methodology and the interpretation of results?	4/4
Is there a statement locating the researcher culturally or theoretically?	1/4
Is the influence of the researcher on the research, and vice-versa, addressed?	1/4
Are participants, and their voices, adequately represented?	4/4
Is the research ethical according to current criteria or, for recent studies, and is there evidence of ethical approval by an appropriate body?	4/4
Do the conclusions drawn in the research report flow from the analysis or interpretation, of the data?	4/4
Quantitative research	
Were the criteria for inclusion in the sample clearly defined?	13/13
Were the study subjects and the setting described in detail?	13/13
Was the exposure measured in a valid and reliable way?	Not applicable
Were objective, standard criteria used for measurement of the condition?	13/13
Were confounding factors identified?	5/13
Were strategies to deal with confounding factors stated?	5/13
Were the outcomes measured in a valid and reliable way?	10/13
Was appropriate statistical analysis used?	13/13

## Data Availability

Not applicable.
